# Groundwater Arsenic Contamination in the Ganga River Basin: A Future Health Danger

**DOI:** 10.3390/ijerph15020180

**Published:** 2018-01-23

**Authors:** Dipankar Chakraborti, Sushant K. Singh, Mohammad Mahmudur Rahman, Rathindra Nath Dutta, Subhas Chandra Mukherjee, Shyamapada Pati, Probir Bijoy Kar

**Affiliations:** 1 School of Environmental Studies, Jadavpur University, Kolkata 700032, India; 2 Virtusa Corporation, Irvington, NJ 07111, USA; sushantorama@gmail.com; 3 Global Centre for Environmental Remediation (GCER), The University of Newcastle, Callaghan Campus, Callaghan, NSW 2308, Australia; mahmud.rahman@newcastle.edu.au; 4 Department of Dermatology, Institute of Post Graduate Medical Education and Research, SSKM Hospital, Kolkata 700020, India; rndutta@gmail.com; 5 Department of Neurology, Medical College, Kolkata 700073, India; drscmukherjee@gmail.com; 6 Department of Obstetrics and Gynecology, Calcutta National Medical College, Kolkata 700014, India; shyamapada_pati@rediffmail.com; 7 Surgical Oncologist, Barasat Cancer Research and Welfare Centre, Kolkata 700124, India; drpbkar@yahoo.co.in

**Keywords:** arsenic, Ganga River basin, drinking water, health effects, cancerous, social issues, mitigation

## Abstract

This study highlights the severity of arsenic contamination in the Ganga River basin (GRB), which encompasses significant geographic portions of India, Bangladesh, Nepal, and Tibet. The entire GRB experiences elevated levels of arsenic in the groundwater (up to 4730 µg/L), irrigation water (~1000 µg/L), and in food materials (up to 3947 µg/kg), all exceeding the World Health Organization’s standards for drinking water, the United Nations Food and Agricultural Organization’s standard for irrigation water (100 µg/L), and the Chinese Ministry of Health’s standard for food in South Asia (0.15 mg/kg), respectively. Several individuals demonstrated dermal, neurological, reproductive, cognitive, and cancerous effects; many children have been diagnosed with a range of arsenicosis symptoms, and numerous arsenic-induced deaths of youthful victims are reported in the GRB. Victims of arsenic exposure face critical social challenges in the form of social isolation and hatred by their respective communities. Reluctance to establish arsenic standards and unsustainable arsenic mitigation programs have aggravated the arsenic calamity in the GRB and put millions of lives in danger. This alarming situation resembles a ticking time bomb. We feel that after 29 years of arsenic research in the GRB, we have seen the tip of the iceberg with respect to the actual magnitude of the catastrophe; thus, a reduced arsenic standard for drinking water, testing all available drinking water sources, and sustainable and cost-effective arsenic mitigation programs that include the participation of the people are urgently needed.

## 1. Introduction

The Ganga River basin (GRB) is a part of the Ganga-Brahmaputra-Meghna (GBM) river basin, draining 1.08 million km^2^ in Tibet, Nepal, India, and Bangladesh; it covers nearly 26% of India’s land mass and is home to a population of over 500 million [1]. Historically, the Hindu civilization most likely originated in the Indus Valley region, and the Indus River was possibly a holy river for this culture [2]. After the decline of the Indus Valley civilization in approximately 1900 BC, people migrated from the east to the Ganga plain and began using Ganga water to supplement their daily needs; at that time, the Ganga was considered a holy river [2]. The GRB is one of the most fertile and densely populated areas in the world, and its inhabitants rely directly or indirectly on this basin for water, food, and agriculture [1]. Devout Hindus still believe that the Ganga’s water will never be polluted and that this holy river is the root of spiritual purification. However, currently available scientific literature reveals that the Ganga is considered to be one of the world’s most polluted rivers, containing a number of toxins including arsenic, cadmium, chromium, copper, lead, and mercury, as well as pesticides and pathogenic microbes nearly 3000 times greater than the safe limit prescribed by the World Health Organization (WHO) [3,4]. Population growth in India continues unabated, and most of the domestic waste of this huge population is dumped in rivers, whether or not the river is considered holy, as well as in various other water bodies [3]. Industrial growth is also increasing, and due to lax regulations, most industries’ untreated waste is dumped into bodies of water. The current waste disposal rate has almost doubled over the last 20 years and is expected to increase by 100 percent in the next two decades. In addition, it is becoming ever more likely that water will not flow from source to sink by way of rivers in the near future. Silt accumulation is decreasing the carrying capacity of all rivers, increasingly resulting in pockets of stagnant waste in most rivers for some portions of the year as can currently be observed in the Yamuna River. Before 1940, any individual Indian of any caste (a system of rigid layering of society associated with the Hindu religion. An endogamous kinship group is the self-perceived “caste” of the individual Hindu, or has been at least until relatively recent times) or tribe would have had difficulty accepting that water from the Ganga River could be polluted and unsuitable for drinking. Over the last 75 years, however, the Ganga has become so polluted that now even a devout Hindu may not believe that Ganga water is safe to drink [3]. Recent reports have maintained that water from the Ganga in the city of Varanasi in the state of Uttar Pradesh is not even safe for bathing [5]. Thus, several questions have arisen regarding why and how this state of affairs has come to pass.

## 2. Reasons behind the Pollution of the Ganga

The water pollution affecting the Ganga is currently anthropogenic [3]. From the annals of history, until 1940, drinking water in India-except in a few major cities-came from streams, ponds, dugwells, lakes, and rainwater. Early available history also makes it clear that Indians preferred Ganga water, if available, for drinking. There was a set idea that the water of the river Ganga could never become polluted. One scientific reason might be that the minerals present in the Ganga’s water prevent bacterial growth. At an earlier time in India, between the time of death and cremation, small quantities of water from the Ganga would be poured into the mouth of the deceased at set intervals until distant relatives could arrive. This ritual might have been an effort to prevent bacterial growth. Even Nobel Laureate Sir Rabindranath Tagore (b. 1861) mentioned in his book Chelebela (childhood) how water from the Ganga River was collected during winter and kept in a large earthen pot for year-round drinking [6]. In the 1950s, India took a step forward by pursuing a green revolution using chemicals and underground water. Population increased unabated, poor sanitation continued, domestic wastes were dumped in rivers, and the number of factories producing synthetic chemicals and dyes mushroomed. Waste generated from their activities and runoff from agricultural fields rich in fertilizers, pesticides, insecticides, herbicides, etc. made traditional sources of drinking water unfit for consumption including the holy Ganga water [3]. Waterborne diseases increased because of the amplified presence of pathogens in surface drinking water. This was the scenario when the WHO and the United Nation’s Children Fund (UNICEF) proposed that underground water from tube-wells should be used for drinking water as it would be relatively free from pathogens. The GRB has plenty of groundwater; as a result, the first tube-wells began to be established in villages in the first half of the 1960s with both national and international assistance. During that time, the WHO and UNICEF, the two primary institutions dealing with health, felt that if the people of the GRB could receive pathogen-free drinking water, the main killers water-borne diseases would be under control. However, the WHO, UNICEF, and other international scientific organizations overlooked the possibility that other chemicals/toxins present in groundwater, such as arsenic and fluoride, could serve as mass killers. The Ganga River system, originating in the Himalayas, carries nearly 15 billion tons of sediments containing arsenic and other trace elements, between 6–16% of the global annual sediment flux [7]. The over-withdrawal of groundwater through hand pumps and redox conditions, briefly discussed in the following sections, have triggered the mobilization of arsenic in the GRB groundwater.

## 3. Current Magnitude of Groundwater Arsenic Contamination in the GRB

India covers 79% of the GRB, affecting numerous states such as Uttarakhand, Uttar Pradesh, National Capital Territory (NCT) of Delhi, Madhya Pradesh, Bihar, Jharkhand, Rajasthan, Chhattisgarh, Punjab, Haryana, and West Bengal. Groundwater arsenic contamination was first reported in 1976 in Chandigarh and in different villages of the Punjab and Haryana states [8]. Five of nine patients, all of whom were ingesting arsenic-contaminated water in the study area, exhibited a high concentration of arsenic in the liver and showed symptoms of non-cirrhotic portal hypertension (NCPH). The author concluded that “cirrhosis (adult and childhood), non-cirrhotic portal fibrosis (NCPF) and extrahepatic portal vein obstruction in adults are widespread in India and suggested consumption of arsenic contaminated water may have some role in the pathogenesis of these clinical states” [9]. However, the significance of this information went unremarked at the time. In 1983, arsenic poisoning was reported in the state of West Bengal [10] and came into the limelight after the International Conference on Arsenic, organized by the School of Environmental Studies (SOES), Jadavpur University, Kolkata, India, brought it into focus [11]. The SOES discovered elevated levels of arsenic in the groundwater of Rajnandgaon district in the state of Madhya Pradesh (now Chhattisgarh) in 1999 [12], in Bihar [13] and Uttar Pradesh in 2003 [14], in Jharkhand in 2004 [15,16], and along the Allahabad-Kanpur track in 2009 [17]. Lalwani et al. testified to finding arsenic levels up to 100 µg/L in the groundwater of Delhi, one of the Union Territories and the capital city of India [18]. Duggal et al. reported arsenic in the groundwater of Rajasthan in four districts: Hanumangarh, Gangapur, Churu, and Sikar. The Sikar alone, as it only partially lies in the GRB, was found to have arsenic levels <10 µg/L [19]. Currently, the groundwater in 25 of 75 districts of Uttar Pradesh, 22 of 38 districts of Bihar, three of 24 districts of Jharkhand, three of 33 districts of Rajasthan, 14 of 22 districts of Haryana, two of 27 districts of Chhattisgarh, one of 11 districts of Delhi, and 14 of 19 districts of West Bengal, is contaminated with arsenic above the WHO standard of 10 µg/L [20]. The other Indian states, including Uttarakhand and Himachal Pradesh, have not been surveyed.

In 1992, SOES first detected arsenic in the groundwater of Bangladesh. While working in various arsenic-affected areas of West Bengal, we observed that women who had migrated from the bordering areas of Bangladesh to West Bengal after marriage had arsenical skin lesions [21]. Over more than two decades, the SOES analyzed 54,000 water samples from tube-wells covering all 64 districts of Bangladesh. Fifty-nine of 64 districts exceeded the WHO limit for arsenic in drinking water, and 50 of 64 districts exceeded the Bangladeshi safe limit of 50 µg/L of arsenic in drinking water with the highest concentration being 4730 µg/L [22].

The Department of Water Supply and Sewerage (DWSS), with assistance from the WHO, first tested the groundwater for arsenic in 1999 in the Jhapa, Morang, and Sunsari districts of Nepal [23]. However, most of the contaminated samples came from the active flood plains of the Koshi River. Later, high arsenic concentrations (up to 170 µg/L) in tested samples were reported in the Nawalparasi, Rautahat, Bara, and Bardia districts. Twelve districts, including Liam, Jhapa, Morang, Udaypur, Mahottari, Parsa, Kathmandu, Lalitpur, Chitwan, Palpa, Dang, and Bardiya have been reported to have arsenic concentrations below the WHO standard. Other districts including Sunsari, Saptari, Siraha, Dhanusha, Sariahi, Rautahat, Bara, Nawalparasi, Rupandehi, Kapilbasu, Banke, Kailali, and Kanchanpur have been reported to have arsenic levels greater than the WHO standard and, in some cases, above the Nepali standard of arsenic (50 µg/L) for drinking water [24].

**Figure 1:**
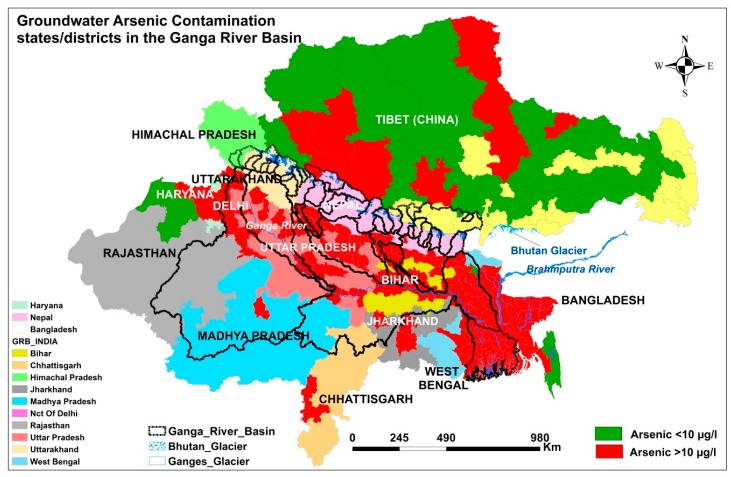
Current status of groundwater arsenic contamination in the GRB.

In Tibet, arsenic contamination was first reported in river water due to wastewater discharge from a geothermal power plant [25]. Wang et al. reported the arsenic concentration (195 µg/L) in a dug well (groundwater) at Xungba, Tibet, arsenic levels up to 5985 µg/L in hot springs, and 10,626 µg/L in alkaline salt lakes in western Tibet [26]. In a recent study, the authors reported an average of 11.6 µg/L of arsenic in shallow wells and only 6.2 µg/L in deep wells; however, average arsenic concentrations were comparatively higher in water samples from hot springs (241.4 µg/L), lakes (27.5 µg/L), and streams (22.1 µg/L) [27]. Figure 1 shows arsenic-contaminated areas in the GRB.

## 4. Sources and Mechanisms of Groundwater Arsenic Contamination in the GRB

Present research indicates that all rivers, susceptible to arsenic contamination through arsenic-containing sediment loads originate in the Himalayan Mountains and the Tibetan plateaus [16]. The reason for the presence of arsenic is in all probably geologic. Various theories have been postulated on the origins of arsenic and its movement from the source point [28,29,30,31,32,33,34]. It is considered that arsenic, which is present in aquifer sediment with iron oxides, has been released by microbially-driven reductive dissolution in an organic-rich environment [29,32,35]. However, the exact nature and mechanisms involved in the movement process are still unknown [35]. In the Chhattisgarh state of India, arsenic groundwater contamination is due to natural deposition of arsenic-rich pyrite, and its mobilization is due to microbial respiration of organic carbon [36].

In Bangladesh, reduction of Fe-oxyhydroxides and the degradation of organic matter are reported to be the arsenic release mechanism in the groundwater [37]. The arsenic-contaminated Terai region of Nepal shares similar geologic characteristic properties to those of the Bengal Delta Plain, and arsenic mobilization in the groundwater in Nepal has been linked to the oxidation of organic matter, microbial activities, and geochemical changes [38]. Limited studies in Tibet have shown that arsenic contamination in various surface and groundwater sources is linked to carbonite abundance, and arsenic levels have demonstrated a westward gradient within the region [27]. One of the biggest challenges in understanding arsenic release mechanisms and predicting groundwater arsenic contamination is the high temporal and spatial variability of arsenic in the GRB, along with diverse geologic and topographic settings. Consequently, aquifer-specific studies are needed [39,40].

## 5. Impacts of Arsenic on Human Health in Chronically Exposed Population in the GRB

The available arsenic-induced health effect reports come mainly from epidemiological studies of chronic arsenic exposure, and inorganic arsenic has been confirmed as responsible for numerous illnesses [41,42]. Arsenic can cause various adverse health effects such as dermal, cardiovascular, respiratory, gastrointestinal, endocrinological (diabetes mellitus), neurological, reproductive and developmental, cancerous, and cutaneous effects, with the latter being the most common in the GRB [43]. The appearance of skin lesions is a red flag, as these are a manifestation of severe internal damage [44]. The irony is that to date, no known medicine can cure chronic arsenic toxicity. Only arsenic-safe water and nutritious food, including vitamins, are suggested as a preventive measure.

### 5.1. Dermatological Effects

Dermatological effects have been diagnosed predominantly in the West Bengal, Uttar Pradesh, Bihar, and Jharkhand states of India and Bangladesh [14,16,43,44]. Dermatological effects in 42% of adults (*n* = 150) and 9% of children (*n* = 58) were also diagnosed in the Rajnandgaon district of Chhattisgarh [12].

**Figure 2:**
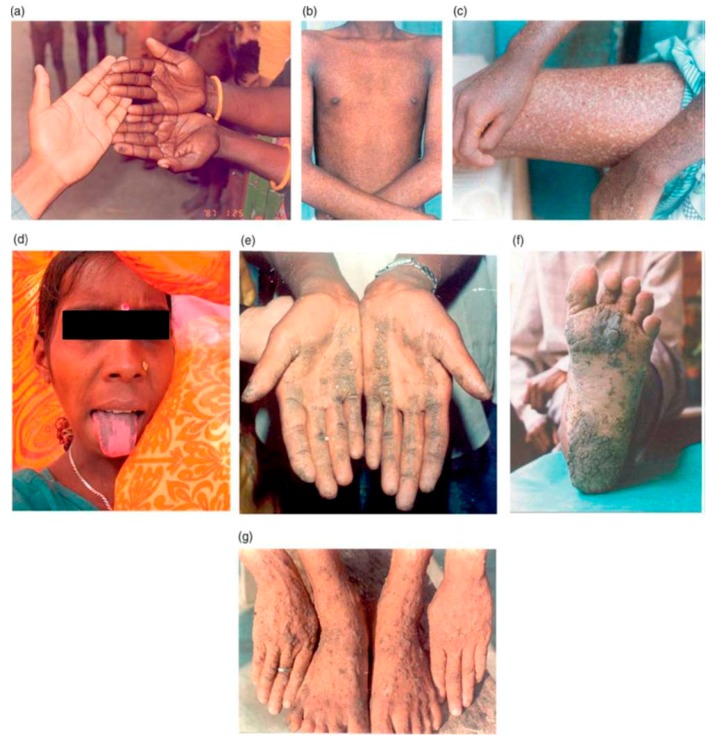
Different skin symptoms due to arsenic toxicity: (**a**) diffuse melanosis; (**b**) spotted melanosis; (**c**) leucomelanosis; (**d**) tongue melanosis; (**e**) diffused and nodular keratosis on the palm; (**f**) spotted keratosis on the sole; and (**g**) dorsal keratosis.

Cutaneous manifestations are the most prominent characteristic used in identifying arsenicosis patients [43,44]. Skin lesions can occur even at 50 µg/L of arsenic in drinking water [45]. However, as per our observations in these Bangladeshi and Indian states, the likelihood of dermal effects is very high if a person consumes ≥300 µg/L of arsenic over a few years’ time [16]. The odds of arsenical dermatosis are high among people over 40 years of age [46]. Normally, diffuse melanosis, a darkening of the skin on the body or palms of the hands, is the earliest symptom (Figure 2a). However, individuals suffering from arsenic toxicity may not always develop diffuse melanosis symptoms [43]. Spotted pigmentation (spotted melanosis) commonly appears on the chest, back, or the limbs and is considered the second stage (Figure 2b). Victims of spotted melanosis develop white and black spots on their bodies, popularly known as Leucomelanosis, after they discontinue the consumption of arsenic contaminated water (Figure 2c) [16,43,44]. Arsenic toxicity may also cause mucous membrane melanosis inside the mouth including on the gums, lips, and tongue (Figure 2d). Diffuse with nodular keratosis on the palm (Figure 2e) and sole (Figure 2f) and spotted keratosis (rough, dry skin with palpable nodules) on the dorsal side of the hands, feet, and legs are signs of severe arsenic toxicity (Figure 2g) [16,43,44]. In Nepal, dermatosis is also reported in 1.3 to 5.1% (*n* = 5215) of surveyed individuals residing in the Bara, Parsa, and Nawalparasi districts where >50 µg/L of arsenic (up to 1200 µg/L) has been detected in the sources of drinking water [38]. Arsenicosis reports from Tibet are not currently available in the literature.

### 5.2. Cardiovascular Effects

Cardiovascular effects such as ischemic heart disease, peripheral arterial disease or “blackfoot disease”, systemic arteriosclerosis, and gangrene have been documented as an independent risk factor for human health induced by prolonged arsenic exposure and are irreversible [46]. Several patients with gangrene of the foot were also diagnosed in West Bengal, India, and Bangladesh. The gangrene-affected legs of the victims had to be amputated. Hypertension, also linked with arsenic exposure, was detected among a majority of the studied individuals in arsenic-affected areas of Bangladesh [46].

### 5.3. Respiratory Effects

Studies conducted in the GRB confirm that chronic exposure to arsenic could lead to various respiratory disorders including cough, shortness of breath, noisy chest while breathing, and even non-malignant as well as malignant lung diseases [16,43]. These symptoms were positively correlated with arsenic concentrations in drinking water that ranged from <3 to 3400 µg/L, among the studied 7683 chronically exposed individuals in West Bengal, India [47]. Studied women were more likely to have a cough, chest sounds, and shortness of breath than men [47]. The supposition that “long-term chronic exposure to arsenic through ingestion can cause respiratory effects”, was reconfirmed in Bangladesh when nonsmokers with skin lesions (*n* = 44) who had been exposed to a high concentration of arsenic (136 to 1000 µg/L) were compared to nonsmoker control groups (*n* = 125) with no exposure to arsenic [48]. Elevated arsenic concentrations can also increase the prevalence ratio of chronic cough and bronchitis [49]. There could be several such groups of victims in other parts of the GRB that have not yet been investigated. Also, the victims may not be aware of arsenic as the cause of existing chronic respiratory symptoms, since these are common health issues in most rural areas, and the existing medical systems are not well trained and equipped for specialized diagnosis.

### 5.4. Gastrointestinal Effects

Mild to severe arsenic-induced gastrointestinal effects are reported in West Bengal India and Bangladesh [16]. Thirty-eight percent (60 of 156) of the surveyed villages, chronically exposed to arsenic-contaminated groundwater in the West Bengal state of India, exhibited chronic or recurrent pain or discomfort in the upper abdomen, commonly known as dyspepsia [50]. In another study in West Bengal, India, individuals in the surveyed communities, chronically exposed to arsenic-contaminated water between 30 and 3400 µg/L, had persistent abdominal pain when compared to the control population exposed to <50 µg/L of arsenic [16]. In Bangladesh, chronically arsenic-exposed individuals displayed symptoms of nausea, diarrhea, anorexia, and abdominal pain [16].

### 5.5. Hepatological Effects

The first case of hepatological effects, noncirrhotic portal fibrosis, was diagnosed in 1976 in nine patients from Chandigarh, India who had been chronically exposed to arsenic through drinking water containing up to 549 µg/L of arsenic [8]. A significant amount of arsenic was detected in the livers of these patients. Later, hepatomegaly was reported in a majority of individuals exposed to high levels of arsenic that ranged from 200 and 2000 µg/L, in West Bengal, India [45]. It is evident that liver enlargement is a common symptom among arsenic-exposed communities in most GRB regions including West Bengal, India, and Bangladesh [16].

### 5.6. Neurological Effects

Predominant distal poly neuropathy and paresthesia or peripheral neuropathy were diagnosed in a majority of the studied populations exposed to elevated levels of arsenic through drinking water in West Bengal, India [16]. Some neurological disorders were also reported in the Bihar and Uttar Pradesh states of India [13,14]. New investigations have identified several patients with various peripheral neuropathy symptoms including limb pain, hyperpathia or allodynia, distal paresthesia and hypesthesia, calf tenderness, distal limb symptom, and diminished tendon reflexes in West Bengal, India [16].

### 5.7. Reproduction and Developmental Effects

It is a medically proven fact that inorganic and methylated species of arsenic in a chronically exposed woman, can invade the placenta and thus may significantly influence the reproductive and developmental processes [44]. However, other confounding factors might contributing to reproductive and developmental failures, such as exposure period and source, the minimum arsenic level required to trigger adverse reproductive occurrences, congenital malformation, repeated childbirth and malnutrition (very common in rural India and Bangladesh), and other unknown factors [44]. Spontaneous abortions, stillbirths, preterm births, low birth weight, and neonatal deaths were more prevalent among the chronically exposed populations in a majority of the studied individuals of West Bengal, Bihar, and Uttar Pradesh states in India and Bangladesh [13,14,16,43,51,52]. All the victims were exposed to arsenic levels up to 1474 µg/L in their drinking water for at least five years. These women displayed a six-fold reproductive failure compared to women exposed to <20 µg/L of arsenic [44]. Although exposure to elevated levels of arsenic has shown a positive trend in terms of increasing the risk of stillbirth among arsenic-affected women, a dose-response study is needed to establish a clear relationship between spontaneous abortion and infant mortality related to arsenic exposure.

### 5.8. Cancerous Effects

Two decades ago, it was believed that arsenic toxicity could only cause skin cancer. Later, the leading global environmental and health protection authorities, such as the US Environmental Protection Agency (US-EPA), the WHO, and the International Agency for Cancer Research (IARC), declared that arsenic could cause skin, lung, liver, urinary tract, bladder, kidney, and other types of cancers [41,42]. Most epidemiological studies report that an exposure to arsenic ≥300 µg/L may cause internal cancers [53,54,55]. Little data is available demonstrating an exposure concentration below a few hundred micrograms per liter. In this context, Smith et al. [56] have affirmed that cancer risk might be on the order of as high as 1 in 100 at 1 L of water intake/day containing 50 µg/L of arsenic. However, in arsenic-affected GRB, an adult male drinks about 4–5 L of water per day [57,58,59] and the acceptable limit of arsenic in drinking water is 50 µg/L. Evidence of arsenic-induced cancers from drinking water largely comes from ecological investigations and a few case-control and cohort studies. Among other cancers, skin cancers comprise the most common health effects and have been widely studied in countries where people rely on drinking and cooking water containing elevated levels of arsenic contamination. Squamous cell carcinoma and multiple basal cell carcinoma are typical arsenic-induced skin cancers, while Bowen’s disease indicates impending skin cancer [16]. The characteristic arsenic-induced skin carcinomas are squamous cell carcinoma and multiple basal cell carcinomas. Mortality rates are low, and those affected may not be in grave danger. However, skin cancer could be an indication of more serious internal types of cancer [16,44].

**Figure 3:**
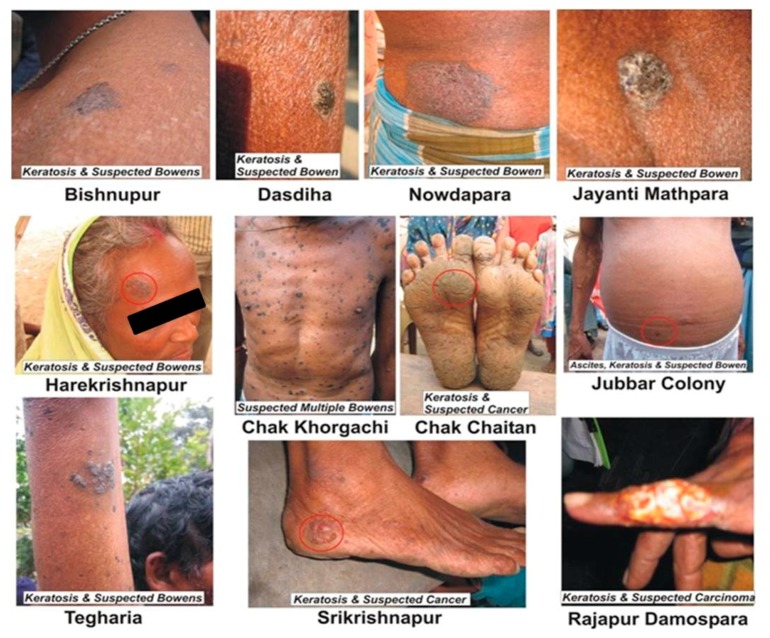
Patients suffering from arsenical keratosis, affected by pre-malignancy.

A preliminary follow-up study on arsenic patients from some arsenic-affected villages of West Bengal and Bangladesh who were suffering from arsenical keratosis, registered from 1995 to 2000, was carried out during 2009–2010. After this study, it was concluded that, on average, 62.5% of patients suffering from arsenical keratosis were affected by pre-malignancy (Figure 3) after ten years.

### 5.9. The Other Health Effects and Future Danger

In addition to the above listed arsenic-induced health effects, several other symptoms for example, diabetes mellitus and immunological disorders have been linked to arsenic in Bangladesh, along with DNA damage in West Bengal [46,60]. Most of these arsenic-affected populations are from a poor socioeconomic background, and the individuals suffer from malnutrition that makes them more vulnerable to the adverse health effects of arsenic contamination [61]. These individuals may or may not have arsenical skin lesions, a common visible symptom of arsenicosis, but they experience loss of appetite, weakness, and lethargy, and they become easily fatigued, which in turn limits their ability to perform physical activities and work, leading to a loss of income. In some cases, sun exposure causes itching skin and burning and watering of the eyes [16,44]. A unique sound, “the cough of arsenicosis”, is another chronic respiratory effect that creates an unusual atmosphere in a neighborhood, especially at night. Moderate to severe anemia, conjunctival congestion, and leg edema were also reported in chronically arsenic-exposed communities. Moreover, during our first 29 years (since 1988) of field surveys with a medical group, we observed that on average, 10% of adults and 2% of children in highly arsenic-affected villages showed arsenical skin manifestations. Around 80% of people in arsenic-affected villages showed an elevated level of arsenic in their biological samples (hair, nails, and urine) indicating they were sub-clinically affected. The appearance of arsenical skin lesions depends on various factors including the concentration of arsenic in the drinking water, amount of water consumption, exposure period, and the nutritional and health status of the person exposed to arsenic.

Over the past decade, the government and other agencies have installed various arsenic-safe sources of water including deep tube-wells, purified surface water, and arsenic removal plants (ARP) in arsenic-affected areas of India and Bangladesh. This is a possible explanation for why new arsenic patients with skin lesions were not detected in these regions, indicating that the people in these villages are not drinking arsenic-contaminated water. In a recent study from the same contaminated areas, the tested drinking water samples did not show a high concentration of arsenic. However, nearly 50% of the underground water and ARP filtered water contains arsenic between 10 µg/L and 50 µg/L, and 10% of water contains between 51 µg/L and 100 µg/L [62]. A small percentage reveals arsenic above 100 µg/L. Although these initiatives have had a significant impact on the reduction of arsenic-induced health risks, 13 out of 1000 individuals may be susceptible to a lifetime risk of dying from various cancers including lung, liver, kidney, or bladder, if they consume 1 L of water per day that is contaminated with 50 µg/L of arsenic [53]. In a similar exposure scenario during early childhood or in utero, arsenic may cause pulmonary effects (both malignant and nonmalignant) that could significantly increase the risk of successive mortality in young adults [55].

### 5.10. Health Effects on Children: Our Future Generations at Risk

**Figure 4:**
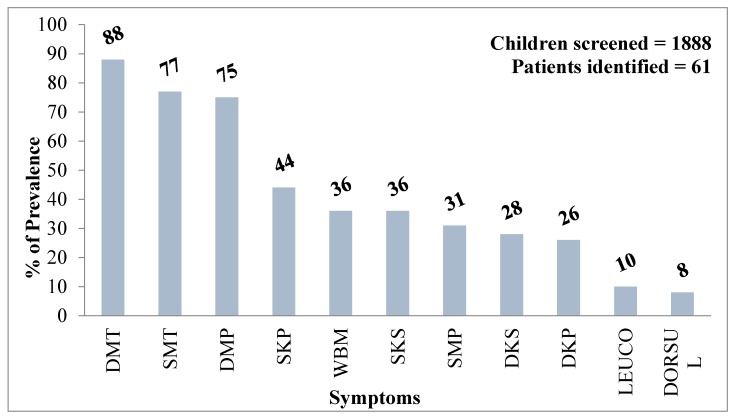
Common dermatological features due to arsenicosis among surveyed children of Bihar, India. Note: DMT: Diffuse Melanosis on Trunk; SMT: Spotted Melanosis on Trunk; DMP: Diffuse Melanosis on Palm; SKP: Spotted Keratosis on Palm; WBM: Whole Body Melanosis; SKS: Spotted Keratosis on Sole; SMP: Spotted Melanosis on Palm; DKS: Diffuse Keratosis on Sole; DKP: Diffuse Keratosis on Palm; LEUCO: Leuco Melanosis; DORSUL: Dorsal Keratosis.

Infants and children show fewer severe arsenical skin lesions than adults; however, they are more vulnerable to the toxic effects of arsenic than adults. A high intake of arsenic through drinking water doubles the likelihood of age-specific health-effects, as the daily water intake per unit of body weight (mL/kg per day) of an infant is three to four times greater than that of an adult. Among more than 130 million Asians exposed to arsenic above the WHO standard of 10 µg/L, an estimated 20 million are less than 11 years of age. In Bihar, India, in our survey of 1888 children of age <11 years, 61 of them showed various symptoms of arsenic skin lesions (Figure 4) [63].

These children drink high concentrations of arsenic (up to 841 µg/L), and in most cases, they are malnourished. Many such groups of arsenic-affected children can only be revealed through detailed epidemiological studies [63].

**Figure 5:**
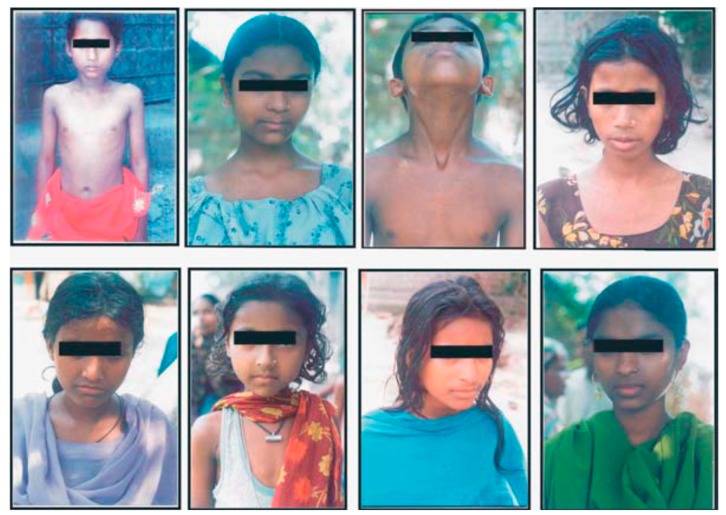
A group of children suffering from arsenical skin lesions in Bangladesh.

Among 7683 individuals studied for arsenic-induced respiratory effects in the West Bengal state of India, 2% of children in the age groups ≤9 and 10–19 demonstrated abdominal chest sounds (crepitations or bronchi), cough, and shortness of breath. Arsenic can significantly impact the central nervous system (CNS) and may lead to short-term memory loss and other cognitive impairments [47]. Recent studies of 301 and 201 children in age groups of 10 and 6 years exposed to arsenic through drinking water in Araihazar Thana of Bangladesh indicated that arsenic consumption can reduce intellectual function, subject to the dosage [64,65]. Figure 5 shows a group of children suffering from arsenical skin lesions in Bangladesh.

## 6. Unsavory Truths: Arsenic in the Food Chain in the GRB

In most developing countries, such as India, groundwater can be exploited for drinking and irrigation purposes without the permission of concerned authorities or the government. Consequently, groundwater resources are used recklessly. Farmers in the country particularly depend on groundwater for cultivating summer paddies. In arsenic-rich areas, tube-wells used for irrigation draw up water replete with arsenic. In reality, they are pumping up poison.

We deduced that nearly 6.4 tons of arsenic could seep into agricultural land through 3200 arsenic contaminated irrigation tube wells in the Deganga block, covering a total area of 200 km^2^ in the North 24-Parganas district of West Bengal, India (Table 1) [66].

A majority (96%) of the tested shallow tube wells contained arsenic above 10 µg/L with an average concentration of 71 µg/L. Each shallow tube well can discharge on average 20 m^3^/h, 20 × 7 m^3^/day (7 h/day each), 20 × 7 × 210 m^3^/year (about 7 months each run), and 20 × 7 × 210 × 1000 L^3^/year = 2.94 × 10^7^ L/year; therefore, the total discharge by the 574 shallow tube wells with arsenic >10 µg/L is 2.94 × 10^7^ × 574 L/year = 1.69 × 10^10^ L/year producing 1.2 tons of arsenic every year (1.69 × 10^10^ × 0.071 mg = 1.2 × 10^9^ mg = 1.2 tons). Consequently, the arsenic dumped in the fields inevitably enters the food chain. Our studies have conclusively proven this revealing that over 76% of the arsenic present in the crops is inorganic [67]. Arsenic first seeps into the roots and finds a path to the grain through the stem. The entry of arsenic into the food chain, therefore, can cause further damage to human health. In most cases, crops grown in arsenic-contaminated areas are sold after harvest in uncontaminated areas. Through this channel, arsenic migrates from arsenic- contaminated areas to uncontaminated areas; for example, the city of Kolkata receives most of its food supplies from neighboring arsenic-contaminated areas. Rice is the staple food of most Southeast Asian populations. Rice grains grown on arsenic-contaminated soil or irrigated with arsenic-laced water have shown elevated levels of arsenic compelling the scientists in this region to press the environmental panic button [68,69]. Animals, especially pets, also become vulnerable as water and straw are laced with arsenic. Considerably higher arsenic concentrations have also been reported in various food materials including luffa (800 µg/kg), brinjal (492 µg/kg), cucumber (399 µg/kg), ladyfinger (375 µg/kg), gourd (268 µg/kg), green gram (174 µg/kg), rice (51 µg/kg), rice husk (22 µg/kg), wheat (27 µg/kg), maize (13 µg/kg), and lentils (15 µg/kg) in the middle-Ganga Plain of India [70,71]. In Bangladesh, arsenic levels in vegetables including arum, pumpkin, coriander, radish, gourd leaves, spinach, red amaranth, arum stem, Indian spinach, arum tuber, beans, papaya, green chilli, and eggplant range between 11 and 464 µg/kg, with the highest concentration in gourd leaves and the lowest in beans [72].

## 7. Socioeconomic, Psychological, and Cultural Implications of Groundwater Arsenic Contamination in the GRB

In this section, we list some examples of the socioeconomic, psychological, and cultural implications of groundwater arsenic contamination in the GRB. Rustamsheikh, aged 45, occupationally a brick-maker, provides a sobering example. A longtime resident of the village-Kadamtala, GP-Katlamari-1, District-Murshidabad, he narrated a shocking and painful account during our visit. In the past, he could make approximately 700–800 bricks per day, monthly earning about $100 on average, sufficient to provide a comfortable life for his family. Over the years, arsenicosis seized him with its initial physical weakness and illness. Gradually, arsenical skin lesions began to appear, just as he had observed in many of his friends who lived in neighboring villages. Tearfully, he told how he could make only 300–350 bricks per day. The poison of arsenicosis has snatched away and shattered the peace that his family once enjoyed. The vacant, hapless look in his eyes made our hearts reel with agony.

This is only a single micro-level example of the impact of chronic arsenic exposure on income. In fact, millions of people are at risk from arsenic. In estimating the loss due to arsenic poisoning, the adverse impact on the Gross Domestic Product (GDP) of the country must be calculated. Nearly 30% of the GRB population living below the poverty line is illiterate. This means that poisoning by arsenic impacts their socioeconomic and health status and, as a result, puts an additional financial burden on the government, leading to massive economic inefficiencies. In Nepal, arsenicosis patients in a lower income group were found to be more likely to encounter economic and social challenges such as difficulties in receiving treatment, lack of medicine in the hospitals, limited access to hospitals, long wait times for receiving treatments, discrimination in service delivery, lack of separate facilities for female patients, and difficulty in buying medicines [38].

**Figure 6:**
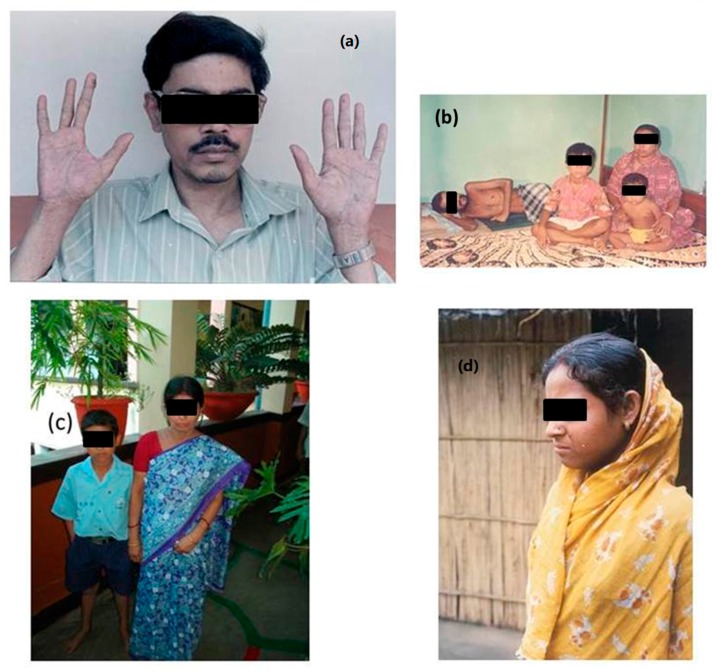
(**a**) Susanta Roy; (**b**) Makhan Pal; (**c**) Bipasha Bhowmik and her son and (**d**) a lady in a village Godagari in Murshidabad.

Arsenicosis victims suffer from social ostracism, social hatred, and enormous psychological trauma. Take the case of Susanta Roy of Jadavpur Narkelbagan in Kolkata, a student of electronics engineering. He had severe arsenical skin lesions, and one of his fingers was amputated because of a cancerous growth (Figure 6a).

Mr. Roy committed suicide as he could not run his business and could not repay a bank loan. About twenty years ago, the corresponding author of this manuscript requested an arsenic-affected college student from the Sarkarpara village of Baruipur block, South 24-Parganas, to appear for a television interview. A deep fear of social isolation was reflected in his letter (S1). Mr. Makhan Pal, a resident of Berachapa village, Deganga block of North 24-Parganas, West Bengal, India, died of cancer when he was 24. Figure 6b shows his letter to us, sent before his death. Figure 6c shows another arsenic patient, Bipasha Bhowmik, and her 10-year-old son Chintu. She is from a village in Sonarpur near Kolkata. Her husband left her when the symptoms of arsenic poisoning became visible on her skin. She used to work as a maidservant but cannot find employment anymore due to her skin lesions.

Figure 6d shows a photograph of a woman in a village who had three failed pregnancies (stillbirth, abortion and an early neonatal death). Arsenic levels in her drinking water and urine were 1617 μg/L and 1474 μg/L, respectively. Due to her reproductive failure, her mother-in-law wanted her son to marry again.

## 8. How to Combat the Present Arsenic Crisis in the GRB

Approximately 90% of the people in the GRB depend on tube-wells for drinking and cooking. Demarcating the arsenic-free and arsenic-contaminated tube-wells by painting them green and red respectively could reduce the arsenic burden immediately, as well as possibly creating awareness among communities. The green tube-wells could be used for drinking and cooking and the red tube-wells for bathing, cleaning, and for the toilet. Installation of new arsenic-safe deep tube-wells in contaminated areas should be ceased until all existing tube-wells are checked for arsenic contamination. India and Bangladesh have ample surface water resources available through rivers, wetlands, and oxbow lakes; therefore, this region is known as the land of rivers. Annual rainfall in these areas is approximately 2000 mm. Bangladesh alone has about 11,000 m^3^ of surface water available per capita. However, uncontrolled massive groundwater extraction has altered the aquifer and has even made the deep aquifer unsafe for domestic needs in these areas. It takes decades to centuries to accumulate a high volume of water in the deep aquifers; it is a gradual process in which rainfall plays a vital role in replenishing the deep aquifer. A rapid depletion of the water level may cause a deleterious influx of arsenic from the shallower arsenic-contaminated layers. Therefore, efficient water management and community participation are crucial in ensuring the sustainable use of these huge water resources. Under continued depletion of the deeper aquifer due to increasing irrigation demands, the current rigorous effort to provide deep tube wells for arsenic-safe drinking water may not achieve sustainability. The existing safe tube wells require monitoring every six months to track the possibility of arsenic contamination as we have discovered that previously safe tube wells in West Bengal now show arsenic contamination [73]. Installation of new tube wells should be strictly regulated. Traditional water management such as dug wells, three-Kalsi arsenic-filtration system, and rainwater harvesting with control over bacterial and other possible chemical contamination could be sustainable solutions in most GRB regions. Additionally, awareness of the arsenic calamity, its associated health risks, and breaking a social tabu that arsenic calamity is not a curse of God is crucial. Area and socioeconomically particular arsenic-free water sources should be provided. For example, in a recent study in the GRB in India, most surveyed individuals assigned high priority to arsenic treatment units (filters) and piped water supply systems and lower priority to deep tube wells, dug wells, and rainwater harvesting systems [74]. This different preference for arsenic-safe drinking water sources depends on arsenic awareness, the willingness to pay for arsenic mitigation technologies, communities’ trust in institutions and their social capital. Therefore, existing institutions and agencies should be reinforced, and communities should be educated about arsenic problems and associated health risks [74]. The author of a recent study has recommended that a cost-effectiveness analysis of existing or proposed arsenic mitigation technologies could help in identifying the most cost-effective options if resources are limited [75]. Moreover, Geographical Information System and Remote Sensing tools and techniques promise to be extremely useful for pro-arsenic-mitigation policies by predicting potential arsenic contamination of areas, and cloud-based decision support systems for arsenic health risk assessment could spread arsenic awareness on a realtime basis, potentially covering a vast population [40,74]. Above all, a worldwide effort by the scientific community and public health organizations should be made focusing on cancer, vascular disease, and other complications of arsenic exposure.

## 9. Challenges Associated with Arsenic Standards in the GRB

The initial acceptable arsenic concentration of 200 µg/L in drinking water set by the WHO in 1958, was based on 2 L of water consumption per day. This figure was further revised to 50 µg/L in 1984, based on available human health data postulating that 200 µg/L of arsenic is not safe to drink, and 50 µg/L would not have any adverse health effects on humans. However, in 1993, the WHO again reduced the guideline value for arsenic in drinking water from 50 µg/L to 10 µg/L as a provisional guideline value, based on available epidemiological studies maintaining that at 50 µg/L, arsenic can cause lifetime cancer and some other health risks. Nonetheless, the WHO recommended that the current arsenic standard be further reduced, depending on possible health effects, socioeconomic and nutritional status, and arsenic mitigation capabilities of a region. A group of scientists opined that the WHO limit of 10 µg/L might not be safe for pregnant mothers and children [76]. Considering health effects, the US states of New Jersey and South Carolina accepted the 5 µg/L level for residences; in Australia, it is 7 µg/L. It has been further reported that ingesting water with just 1 µg/L of arsenic for a long time can cause endocrine disruption including cancerous tumors, congenital disabilities, and other developmental disorders [76,77,78]. In most cases, in the GRB, the arsenic levels in drinking, cooking, and irrigation water is several times higher than that which is detectable in other parts of the world. Moreover, arsenic-affected communities live in poverty and harbor scarce nutritional foods; therefore, the human question arises: Is the WHO standard of 10 µg/L of arsenic for drinking water safe for the millions in impoverished populations? Considering the cancer risks coupled with the compounding factors of poor nutrition for many arsenic-exposed individuals in India, in September 2003, the Bureau of Indian Standards (BIS) recommended [79] the guideline value of arsenic by the WHO (10 µg/L) as the drinking water standard for India. While the scientific community congratulated the BIS for this decision, the revised arsenic standard for drinking water did not stand for long after a group of scientists suggested raising the arsenic standard to 50 µg/L for India and other developing countries. Resource constraints and the possible slowing of short-term solutions under a stricter arsenic standard were held responsible for this increase in the arsenic standard. Consequently, the Indian arsenic standard for drinking water reverted to 50 µg/L [56]. However, the SOES debated that considering the severity of potential arsenic-induced health effects, even at concentrations below 50 µg/L, a sincere effort should be made to overcome resource limitations [80]. Although the change from 10 µg/L to 50 µg/L was suggested in 2003 to last a short period, it remained unchanged by the BIS after 6 years (a unique example of negligence); thus, in 2009, drinking water standards were finally revised [81], listing 10 µg/L for arsenic as the desirable level. However, the legally enforceable standard was set at 50 µg/L in the absence of alternative arsenic-safe sources of drinking water. This decision of the BIS is scientifically meaningless. In case of India, this decision means that an individual can “safely” drink water with up to 50 µg/L of arsenic-contamination. Will the scientific community accept this decision of the BIS?

**Figure 7:**
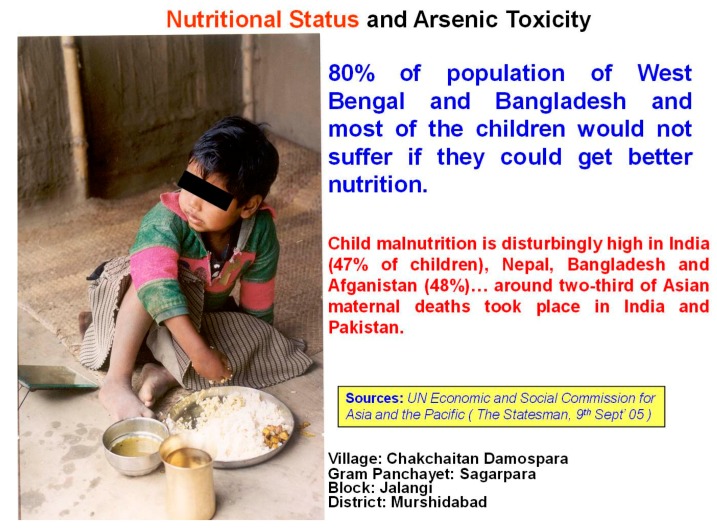
Malnutrition.

The SOES reported [57] that in tropical countries, the average daily direct intake of water for adults is around 4 L; however, the WHO limit of 10 µg/L is based on an estimate of 2 L water intake per day. The WHO guideline value also did not consider indirect water intake. Total water consumption (direct + indirect) for adults in arsenic-affected areas of the GRB is about 6 L per day [57]. It was also reported that better nutrition could help individuals resist arsenic toxicity [16]. In the arsenic-affected GRB, 80% of the population suffering from arsenic toxicity also suffers from malnutrition. Figure 7 shows an example from our study.

The irony is that the states and countries located in the GRB still follow the guideline of 50 µg/L of arsenic as the standard for drinking water. Considering the permissible levels of arsenic in drinking water in developed and developing countries, the question must be raised: Are some animals more equal than others?

## 10. Conclusions

The GRB is highly vulnerable to groundwater arsenic contamination. Elevated levels of arsenic in drinking and irrigation water, and in the food materials are reported in many places in this region. Numerous people demonstrated dermal, neurological, reproductive, cognitive, and cancerous effects and many arsenic-induced deaths of youthful victims are testified in the arsenic contaminated areas of the GRB. Arsenic victims also encounter critical social and economic challenges in the form of social isolation and hatred by their respective communities and loss of jobs, respectively. Failure of arsenic mitigation programs and the lack of community’s participation have aggravated the arsenic catastrophe in the GRB and put millions of lives in danger.

## Figures and Tables

**Table 1:** Distribution of arsenic in water from irrigated tube wells in Deganga block, North 24 Parganas, West Bengal, India.

Total No. of Irrigated Tube Wells	Total No. of Irrigated Tube Wells Analyzed	No. of Water Samples Having Arsenic (μg/L)		Distribution of No. of Samples in Different Concentration Range (μg/L) of Arsenic							
		>10	>50	<10	10–50	51–99	100–299	300–499	500–699	700–1000	>1000
3200	597	574	234	23	339	118	98	11	5	2	--
